# Prognostication in Pulmonary Arterial Hypertension with Submaximal Exercise Testing

**DOI:** 10.3390/diseases3010015

**Published:** 2015-02-06

**Authors:** Vinod Khatri, Jennifer E. Neal, Charles D. Burger, Augustine S. Lee

**Affiliations:** 1 Pulmonary and Critical Care Medicine, Mayo Clinic, 4500 San Pablo Road, Jacksonville, FL 32224, USA; E-Mails: Khatri.Vinod@mayo.edu (V.K.); Lee.Augustine@mayo.edu (A.S.L.); 2 Northwestern University Feinberg School of Medicine, Chicago, IL 60611, USA; E-Mail: Jennifer.neal@northwestern.edu

**Keywords:** submaximal exercise test, pulmonary artery hypertension, REVEAL registry, cardiopulmonary exercise test, V_E_/V_CO2_

## Abstract

Introduction: The submaximal exercise test (SET), which gives both a measure of exercise tolerance, as well as disease severity, should be a more robust functional and prognostic marker than the six-minute walk test (6MWT). This study aimed to determine the prognostic value of SET as predicted by the validated REVEAL (Registry to Evaluate Early and Long-Term Pulmonary Artery Hypertension Disease Management) registry risk score (RRRS). Methods: Sixty-five consecutive patients with idiopathic and associated pulmonary arterial hypertension (PAH) underwent right-heart catheterization, echocardiogram, 6MWT and a three-minute SET (Shape-HF™). Analyses explored the association between SET variables and prognosis predicted by the RRRS. Results: Although multiple SET variables correlated with the RRRS on univariate analyses, only V_E_/V_CO2_ (ρ = 0.57, *p* < 0.0001) remained an independent predictor in multivariate analysis (β = 0.05, *p* = 0.0371). Additionally, the V_E_/V_CO2_ was the most discriminatory (area under receiver operating characteristic curve, 0.84) in identifying the highest-risk category (RRRS ≥ 10), with an optimal cut-off of 40.6, resulting in a high sensitivity (92%) and negative-predictive value (97%), but a lower specificity (67%). Conclusion: SETs, particularly the V_E_/V_CO2_, appear to have prognostic value when compared to the RRRS. If validated in prospective trials, SET should prove superior to the 6MWT or the RRRS, with significant implications for both future clinical trials and clinical practice.

## 1. Introduction

Identification of prognostic factors that affect survival has been a key goal in the clinical care of patients with pulmonary arterial hypertension (PAH). The REVEAL (Registry to Evaluate Early and Long-Term Pulmonary Artery Hypertension Disease Management) cohort helped identify some important independent predictors, as well as a composite scoring system—the REVEAL registry PAH risk score (RRRS)—to help in prognosticating these patients [1,2]. The REVEAL study confirmed the increased risk of mortality in patients with World Health Organization (WHO) Group I PAH, including those with portal hypertension [3] and connective tissue diseases [4,5].

The elements of the RRRS are readily available and include the patient’s WHO functional class (WHO-FC), echocardiographic and hemodynamic parameters, as well as the six-minute walk test (6MWT) as a marker of exercise tolerance. Although the 6MWT is considered a simple, noninvasive, inexpensive marker of functional status and exercise tolerance, it suffers from several limitations, including learning effect, day-to-day variability and anthropomorphic/demographic variables [6]. Additionally, the factors that go into the 6MWT include non-cardiopulmonary factors that have little to do with PAH or right ventricular function (e.g., neurologic disorders, musculoskeletal issues, peripheral arterial disease, conditioning, effort). Despite these and other limitations, the 6MWT has been widely used as an important clinical endpoint of most PAH treatment trials and, thus, has been used regularly in the clinical monitoring and management of PAH patients. More recently, a meta-analysis showed that changes in the six-minute walk distance (6MWD) may not correctly predict favorable clinical outcomes [7].

In comparison, the cardiopulmonary exercise test (CPX) provides more comprehensive evaluation of exercise tolerance, but has more specific measures to evaluate ventilation, gas exchange, cardiac function, as well as muscle physiology and, therefore, could be more pertinent in the management of patients with PAH. Specific to PAH, both peak oxygen consumption (V_O2_) and ventilatory equivalent of carbon dioxide (V_E_/V_CO2_) obtained from a CPX appear to be important predictors of survival [8,9,10]. Some investigators have suggested using CPX variables as target goals of therapies (e.g., V_O2_ > 15 mL/min/kg and V_E_/V_CO2_ > 55) [6]; however, the routine use of CPX in PAH patients clinically is limited by the added equipment, time and expertise required, and it may not be suitable for the more severe PAH patients with right heart failure at risk for syncope and arrhythmias.

A novel compromise on the benefits and drawbacks of both the 6MWT and the formal CPX is the submaximal exercise test (SET), which is a low-intensity, three-minute exercise test that is easier to perform than a CPX test, yet unlike a 6MWT, is able to acquire some of the key ventilatory variables that can additionally inform the clinician on the status of the cardiopulmonary system [11]. In particular, some of the key SET variables that can be acquired and that have been shown to be perturbed in PAH include lower partial pressures in the end tidal CO_2_ (PET_CO2_), a greater V_E_/V_CO2_, a reduced oxygen saturation and V_O2_ efficiency slope [10,12,13,14,15]. Our center has extensive experience with this tool clinically and has shown that the SET was a more robust marker of PAH severity than the 6MWT [16].

Such a tool that can measure functional status, exercise tolerance and more specific hemodynamic/ventilatory markers may not only help in prognostication, but may also potentially be an important surrogate endpoint in and of itself in clinical trials and, eventually, in clinical practice, to guide the management of PAH patients. Towards this eventual goal, we first sought to determine whether the SET variables would correlate with the validated RRRS as a prognostic marker, particularly in identifying high risk patients for whom management would be escalated.

## 2. Experimental Section 

Consecutive WHO Group 1 PAH patients seen by the Pulmonary Hypertension Clinic between March, 2011, and May, 2013, were eligible for the study. All subjects had received a right heart catheterization, an echocardiogram a 6-minute walk test with its distance (6MWD), WHO-FC, as well as a SET.

The Shape-HF™ (Shape Medical Systems, Inc.) was utilized for SET in this study. Specific measures able to be obtained by this test include: (1) V_E_/V_CO2_, a measure of breathing efficiency defined as the linear slope of the amount of air expired per minute (V_E_) *versus* the amount of carbon dioxide produced per minute (V_CO2_); (2) the oxygen uptake efficiency slope (V_O2_/log V_E_), indicating the amount of oxygen that is used per unit ventilation; (3) PET_CO2_, reflecting the alveolar CO2 partial pressure at the end of expiration; (4) the heart rate decay during the first minute of exercise recovery; and (5) the chronotropic response index, a measure of the patient’s heart rate response to dynamic exercise. In this study, we specifically examined whether the peak V_O2_ (mL/kg/min), V_E_/V_CO2_ and the partial pressure of carbon dioxide at baseline and at the end of exercise (PET_CO2-b_, PET_CO2-ex_) would correlate with the RRRS.

Baseline characteristics are provided with descriptive statistics. For consistency, medians with their interquartile ranges (IQR) are reported for continuous variables. Non-parametric Spearman’s correlation (ρ) was computed between the raw numeric RRRS and the SET variables. A multivariate analysis to predict the RRRS from the SET variables of age, sex, V_E_/V_CO2_, PET_CO2-b_ and the PET_CO2-ex _was also performed to identify significant predictors of the prognostic risk based on the RRRS. Finally, to identify a potential SET variable to distinguish those with the worst prognosis (RRRS ≥ 10), we performed univariate analyses with non-parametric assumptions (Mann–Whitney test) comparing each of the SET variables between those RRRS ≥ 10 to those with RRRS < 10. We then explored potential cut-offs for the SET variable V_E_/V_CO2_ (which was the most discriminatory) by constructing a receiver-operating characteristic (ROC) curve and its area under the curve (AUC), as well as determining the sensitivity, specificity, predictive values and likelihood ratios (LHR) for the optimal cutoff. All statistical analyses were performed using JMP® Pro 9.0.1 (SAS, Cary, NC, USA). The study was approved and overseen by the Mayo Clinic Institutional Review Board (IRB 12-003335).

## 3. Results and Discussion 

### 3.1. Results

Sixty-five subjects were enrolled and available for analysis. The majority were older women, with an even separation of associated PAH (APAH) from idiopathic PAH (IPAH). On average, most had moderate to severe pulmonary hypertension and were at least WHO-FC III. The specific baseline characteristics are presented in Table 1. There were no adverse events noted during any of the SET.

**Table 1:** Baseline characteristics.

	N = 65
Demographics	
Age, years, median [IQR]	62 [50–70]
Sex, % male	20%
WHO diagnostic group	
IPAH, %	52%
APAH, %	48%
WHO functional class, %	
I	9%
II	35%
III	52%
IV	3%
Right heart catheterization	
mPAP, mmHg	48 [39–57]
PVR, Woods unit	8.5 [4.8–14]
RAP, mmHg	8 [5–11]
PAOP, mmHg	13 [9–16]
CI, L/min/m^2^	2.4 [1.8–3.6]
Other clinical measures	
6MWD, m	367 [284–450]
BNP, pg/mL	82 [38–246]
DLCO, %	59% [45–69]
REVEAL registry risk score	
Median [IQR]	7 [5–9]
Highest risk (≥10), %	20%
Submaximal exercise test	
V_O2_, mL/kg/min	11 [8.8–13]
V_E_/V_CO2_	39 [29–47]
PET_CO2-b_, mmHg	33 [29–36]
PET_CO2-ex_, mmHg	34 [26–37]

IPAH, idiopathic pulmonary arterial hypertension (PAH); APAH, associated PAH; mPAP, mean pulmonary artery pressure; PVR, pulmonary vascular resistance; RAP, right atrial pressure; PAOP, pulmonary artery occlusion pressure; CI, cardiac index; 6MWD, six-minute walk distance; BNP, brain natriuretic peptide; DLCO, diffusion capacity of lungs for carbon monoxide; PET_CO2_, pressure in the endtidal CO_2_.

In univariate analyses, all variables obtained from the SET were significantly correlated with the RRRS; see Table 2. The strongest correlation was modest and seen with V_E_/V_CO2_ (ρ = 0.57, *p* < 0.0001). In a multivariate model with the covariates of age, sex, V_O2_, V_E_/V_CO2_, PET_CO2-b_ and PET_CO2-ex_, the only SET variable that remained an independent predictor was V_E_/V_CO2_ with an effect size of a 0.5 increase in the RRRS for every 10 increase in the V_E_/V_CO2_ (β = 0.045, *p* = 0.0371).

**Figure 1:**
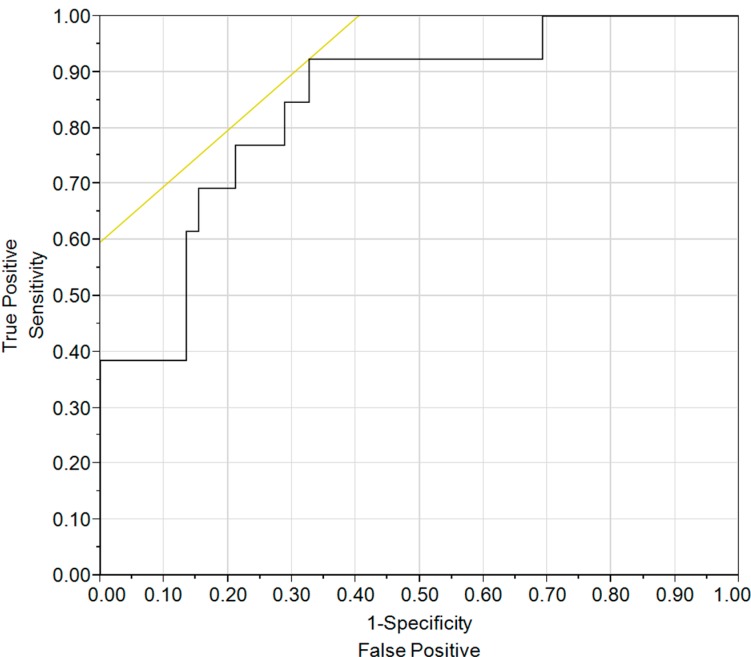
Receiver operating characteristic curve for VE/VCO_2_ in predicting the highest risk category (RRRS ≥ 10). The AUC was 0.84.

Of the 65 patients, 13 (20%) had the worst prognosis as defined by a REVEAL registry score ≥10. The SET variables were compared between these two risk categories. All SET variables proved significantly worse for those in the higher risk category; Table 3. V_E_/V_CO2_ was the most discriminatory among the SET variables. An ROC curve was generated revealing modest discriminatory ability with an AUC of 0.84; see Figure 1. An optimal V_E_/V_CO2_ cut-off of 40.6 revealed a sensitivity of 92% and a specificity of 67%. The positive likelihood ratio or the positive predictive value is poor, but the V_E_/V_CO2_ appeared to have a strong negative predictive value or a negative likelihood ratio; see Table 4.

**Table 2:** Correlation between submaximal exercise test (SET) parameters and the REVEAL registry risk score (RRRS).

SET Variables	Spearman correlation, r	*p*-value
V_O2_	−0.51	<0.0001
V_E_/V_CO2_	0.57	<0.0001
PET_CO2-b_	−0.28	0.0002
PET_CO2-ex_	−0.45	0.026
Delta^*^ PET_CO2_	−0.41	0.0007

* Difference from exercise to baseline. Units are otherwise defined in the text and Table 1.

**Table 3:** SET parameters comparing the highest risk group (RRRS ≥ 10) *versus* the lowest risk group.

SET Variables Median [IQR]	Highest Risk	Lower Risk	*p*-value
	(RRRS ≥ 10)	(RRRS < 10)	
	N =13	N = 52	
V_O2_	9 [6–11]	11 [10–14]	0.005
V_E_/V_CO2_	48 [43–83]	35 [28–43]	0.0002
PET_CO2-b_	30 [27–34]	34 [30–36]	0.033
PET_CO2-ex_	25 [19–33]	34 [30–38]	0.0023
Delta PET_CO2(ex-b)_	−3 [−7.7 to −0.9]	0.9 [−4 to 2]	0.0044

Units are as defined in the text and Table 1.

**Table 4:** Operating characteristics of V_E_/V_CO2_ in predicting the highest risk category (RRRS ≥ 10). LHR, likelihood ratio.

V_E_/V_CO2_	Positive	Negative	Positive	Negative
Cutoff	Predictive Value	Predictive Value	LHR	LHR
29.9	0.27	1.00	1.44	0.00
40.6	0.41	0.97	2.82	0.11
42.7	0.42	0.93	2.86	0.32

### 3.2. Discussion

The current study showed a statistically significant correlation among SET and PAH prognosis as predicted by the RRRS. On univariate analysis, V_E_/V_CO2_ showed the strongest correlation with RRRS and was the only independent parameter that remained significant in multivariate analysis. V_E_/V_CO2_ also had excellent discriminatory power at identifying patients with the highest mortality risk (*i.e.*, RRRS ≥ 10). In particular, the V_E_/V_CO2_ demonstrated excellent sensitivity with high negative predictive value, such that a normal or low V_E_/V_CO2_ value (<40.6) would exclude those least likely to suffer disease progression and death.

Formal CPX testing is a powerful diagnostic and prognostic tool for a variety of cardiopulmonary disorders, including PAH [17,18]. Exercise limitation in PAH is characterized by impairment of oxygen transport and inefficient gas exchange with very high ventilatory demands [19]. Typically, the oxygen transport abnormalities are reflected by a moderate to severe reduction in the peak V_O2_, work rate (WR), anaerobic threshold (AT), V_O2_/WR slope, oxygen (O_2_) pulse and a steep heart rate (HR)-V_O2_ response, combined, on occasion, with a submaximal HR response. The gas exchange response is more conspicuous: high ventilatory equivalents (V_E_/VC_O2_ and V_E_/V_O2_) and a low PET_CO2_, along with exercise-induced hypoxia with advancing disease [15]. Of these parameters, most studies have evaluated, in particular, the prognostic value of the peak V_O2_ [8,9,10,20,21] and, to a lesser extent, the V_E_/V_CO2_ at AT [10,13]. Both of these values require that the patient exercise to the point of achieving AT and maximal exercise. However, in the routine management of a PAH patient, despite being an informative non-invasive test, CPX is infrequently used diagnostically, and rarely repeatedly, to track disease and guide management. This may be, in part, because of the generally sicker PAH population, as well as the additional expertise, time and equipment needed to perform this reliably and safely. Although peak V_O2_ and the V_E_/V_CO2_ at AT may not be obtained during a submaximal test, an elevated breathing efficiency (V_E_/V_CO2_ slope), which can be determined from submaximal data [22] (thus, independent of patient effort and motivation), has been shown to be an important prognostic marker [2,23,24]. As such, SET is an attractive compromise to the limitations of both the CPX and the 6MWT, by providing information on both disease severity and exercise tolerance and, thus, prognosis.

Although previous studies have not specifically evaluated the role of SET in PAH prognosis, there have been few and limited investigations illustrating the potential role of SET in PAH. Woods *et al.* [11,15] recently evaluated the clinical utility of SET in PAH patients and concluded that the various gas exchange variables obtained during the test could differentiate PAH patients from healthy controls and among different severities of PAH. The authors concluded that SET gas exchange may be a useful end point measure in PAH patients.

Adding to this limited body of literature, we found in this study that when compared to the validated RRRS, the SET also provides prognostic value. Given the advantages of the SET over the 6MWT, this supports the need for further exploring SET as an objective tool to be used in clinical trials (for example, as an endpoint in lieu of the 6MWT) or clinically, to guide the clinician in helping to decide who requires treatment or escalation of therapies among the growing complexity of PAH medications now available.

There are significant limitations to consider when interpreting the results of this study. This was an observational, single center study at a tertiary academic center with a specific PAH clinic. Generalizability is thus limited. Furthermore, although there were no adverse events, this may be limited by the small sample size and the selection bias introduced by the clinical selection of PAH undergoing SET testing. However, by practice protocol, all suspected or diagnosed PAH patients undergo SET testing. More importantly, the ability of the SET to prognosticate was obtained, not prospectively, but through a cross-sectional comparison of the SET to a validated prognostic score, the RRRS. We did not have enough long-term follow-up to directly compare the two tools, but our findings have encouraged us to prospectively collect data (ongoing) to more directly determine whether SET will prove a superior marker of both prognosis and disease severity. Despite some of these and other limitations, the biologic plausibility, that a tool that is able to measure both exercise tolerance and disease severity should be a powerful prognostic marker, supports that the findings in this observational study is real. The consecutive collection of hemodynamically confirmed PAH and the systematic characterization of our PAH cohort are additional strengths of this study.

In summary, we are encouraged to further explore prospectively the value of SET in both predicting prognosis, as well as in guiding the management of patients affected by PAH. Furthermore, given the intrinsic advantages of the SET over the 6MWT, we hope future studies will also consider its role as a principle outcome as potentially a superior surrogate biomarker than the 6MWT. If it proves to be a more robust prognostic marker, it may have significant implications, not just for clinical practice, but on the conduct, cost and efficiency of completing clinical trials.

## 4. Conclusions

In this hypothesis generating study, SET variables correlated significantly with the RRRS. Further prospective investigations should be considered to confirm whether SET does have significant prognostic information and to test whether it might prove to be a superior surrogate marker to the 6MWT and/or the RRRS. 
